# Intralesional *Candida albicans* antigen versus intralesional zinc sulfate in treatment of cutaneous warts

**DOI:** 10.1007/s00403-022-02499-w

**Published:** 2022-12-26

**Authors:** Eman M. Kamal Youssef, Maha A. A. Eissa, Radwa M. Bakr

**Affiliations:** 1 Department of Dermatology, Faculty of Medicine, Assiut University, Asyût, Egypt; 2 Tahta General Hospital, Sohag, Egypt

**Keywords:** Immunotherapy, Warts, Candida, Zinc, Intralesional

## Abstract

Immunotherapy represents a promising therapeutic option for treatment of warts. Different concentrations of Candida antigen (1/100 and 1/1000) and zinc sulfate 2% were not previously compared regarding their efficacy in treatment of cutaneous warts. The present study compared the safety and efficacy of intralesional candida antigen versus intralesional 2% zinc sulfate for treatment of cutaneous warts. This prospective controlled clinical trial included one hundred and five patients presented with common, plantar, and plane warts. Patients were divided randomly into three groups, each group included 35 patients. Group 1 were treated with intralesional candida antigen (Ag) 1/100, Group 2 were treated with intralesional candida Ag 1/1000, and Group 3 were treated with intralesional zinc sulfate 2%. This study found that target warts of group 1 displayed higher rate of complete clearance compared to group 2 and group 3 (94.3%, 77.1, 74.2%), respectively. The present study concluded that intralesional immunotherapy with Candida antigen was more effective than Intralesional 2% zinc sulfate in treatment of cutaneous warts and less painful*. *Clinical trial registration number is (Clinical Trials.gov Identifier: NCT03158168).

## Introduction

Two therapeutic options are used for treatment of warts: the first is the destructive method, like chemical cautery, cryotherapy, electrocauterization, surgical excision, and laser ablation, which are painful and with common recurrence [1]. The second is immunotherapy, which depends on the activation of the immune system to suppress virus activity. Such therapy may be applied topical, intralesional injection, or systemic administration [2].

Intralesional (IL) immunotherapy with different skin test antigens like Candida, mumps, or trichophyton antigen induce a delayed type of hypersensitivity response to various antigens and the wart tissue leading to production of Th1 cytokines which activate cytotoxic and natural killer cells to eradicate HPV infection. This clears not only the local warts but also distant warts [3, 4].

Zinc is important for immune regulation as it stimulates the leucocytes and natural killer cells, zinc was found to be deficient in patients with multiple or recurrent warts [5]. Zinc is used in treatment of warts in many studies either topical or systemic [6], while few studies used intralesional [7, 8].

Different concentrations of Candida antigen (1/100 and 1/1000) and zinc sulphate were not previously compared regarding their efficacy in treatment of warts. This study compared the efficacy and safety of intralesional (IL)injection of different concentrations of Candida Ag (1/100 and 1/1000) and zinc sulfate 2% in patients with multiple palmar, plantar, and plane warts.

## Study design

This randomized controlled prospective clinical study (simple randomization) was approved by the Institutional Ethics and Research Committee of Faculty of Medicine, Assiut University, Assiut, Egypt, with clinical trial registration number (Clinical Trials.gov Identifier: NCT03158168). A written informed consent was obtained from each patient or their guardians in case of children, after informing him/her about the technical and scientific basis of the research, the steps of the procedure, and the expected effects or possible complications.

## Patients and methods

A total of 105 randomly selected patients, presented with multiple palmar, plantar, and/or plane warts, were enrolled in the study from March 2018 to March 2020 and completed the study. They were recruited from the Outpatient Clinic of Dermatology Department, Assiut University Hospital.

### Inclusion criteria

Patients with cutaneous (extragenital) viral warts (single or multiple, new, or recurrent, common, plane and plantar warts) of different sizes and durations and with or without distant warts (warts in different anatomical sites) were included. There is no concurrent systemic or topical treatment for warts. Age ranged from 4 to 50 years.

### Exclusion criteria

Patients on any treatment modality for warts at least 1 month before the start of the study. Pregnant or lactating females; patients with immunodeficiency, diabetics, or liver disease were excluded. History of hypersensitivity to *C. albicans* antigen or any of the drugs used for intralesional injection.

All patients included in the study were subjected to:

1. Full history taking including (name, age, sex, occupation, and residence).
2. General and local examinations including (site, number, type of warts, the presence or absence of distant warts, and size of the largest (mother one).

The 105 patients were randomly divided into three treatment groups:

*Group1* (35 patients)—were treated with intralesional (IL) injection of *Candida antigen with 1/100 concentration.*

*Group 2* (35 patients)—were treated with IL injection of *Candida antigen with 1/1000 concentration*.

*Group 3* (35 patients)—were treated with IL injection of *2% zinc sulfate concentration.*

*Candida antigen preparation and storage* Candida Ag solutions, 1/100 and 1/1000, were prepared at Immunity Unit, Ain-Shams University Hospital, Cairo, Egypt. Candida Ag multidose vial was usable for 6 months up to one year and should be stored at cold temperature.

Zinc sulfate 2% preparation: A measure of 2 g of zinc sulfate powder was dissolved in 100 ml of sterile distilled water and autoclaved at 95 C for 20 min [8]*.*

*Treatment procedures*: First, skin was sterilized with 70% ethanol as an antiseptic agent before injection. All the three treatment groups were injected at a dose of (0.1 ml) with a built-in insulin syringe directly in the largest wart (target wart) as a single injection every session. In group 1 and 2*,* no pre-sensitization was done [9]. In group 3, the solution was injected until blanching or bleb formation or until completion of the dose. Subcutaneous injection and acral parts such as fingers and toes were avoided, as it may cause vascular necrosis [8]. All included patients were instructed to wait in the clinic for 15–30 min after injection to observe signs and symptoms of immediate hypersensitivity. Injections were repeated for all patients into the same lesion (largest wart) every two weeks until complete clearance or for a maximum of six treatment sessions. Regarding plane warts in all groups, the same dose (0.1 ml) was divided into multiple injections into multiple warts.

Post Procedure Care for Patients in all Groups:

1. Topical antibiotics and analgesic anti-inflammatory drugs were prescribed to the patients to guard against infection and to relieve pain.
2. All patients were examined after two weeks for assessment of improvement or occurrence of complications.

Follow-up of all patients were done every two weeks for a maximum of six treatment sessions. After the end of the treatment sessions, follow-up was done monthly for at least 6 months to detect recurrence.

*Evaluation* Digital photography was taken before treatment, every treatment session, and monthly for 6 months after completion of sessions using 20-megapixel Sony DSC-W800 digital camera (Sony, Tokyo, Japan). Patients were evaluated for resolution of treated wart (by measuring its diameter with a ruler) and distant warts, reduced size, and number of warts. Photography was done at the same sites of lesions, at the same distance, and under the same illumination using the same camera. Photos were assessed by at least two expert dermatologists blinded of the study procedure in addition to the main study researchers*.* Immediate and late adverse effects were also evaluated after each treatment session.

Clinical response was graded into: *Complete response* (complete cure; *The response was considered complete If there was disappearance of the wart (s) and return of the normal skin markings*), *Partial response* (if warts had regressed in size or number by 50%- 99%), and no *or poor response* (decrease in wart size or number by (0–49%) [10].

Percentage of improvement was calculated as follows:

$$\frac{{\left( {{\text{Size or number before treatment}} - {\text{size or number after treatment}}} \right)\;{\text{multiplied by 1}}00}}{{\text{size or number before treatment}}}$$

*Patient satisfaction* Patients were asked to grade their satisfaction with the therapy by using a quartile grading system. (0 unsatisfied, 1 slightly satisfied, 2 satisfied, or 3 very satisfied) [11]*.*

### Statistical analysis

Data entry and data analysis were done using SPSS version 22 (Statistical Package for Social Science). Data were presented as number, percentage, mean, median, and standard deviation. Chi-square test was used to compare qualitative variables. Mann–Whitney test was used to compare quantitative variables between two groups and Kruskal–Wallis test for more than two groups. Spearman correlation was done to measure correlation between quantitative variables. *P*-value considered statistically significant when *P* < 0.05.

## Results

One hundred and five patients presented with common, plantar, and plane warts were included in the study, their age ranged from 4 to 49 years, 54 were females and 51 were males. New warts were detected in 56 patients, while recurrent warts were observed in 49 patients. The duration of appearance of warts ranged from (3 months–6 years). There was no statistical difference between the three treatment groups regarding their clinical data before treatment except for previous treatment as shown in Table 1. Statistically significant differences were observed between all three groups (*P* = 0.029*) and groups 1 and 3 (*P* = 0.028*) regarding receiving previous treatment modalities as shown in Table 1.

**Table 1:** Clinical data of the studied groups

Clinical data	Group I (*n* = 35)		Group II (*n* = 35)		Group III (*n* = 35)		*P*1-value	*P*2-value	*P*3-value	*P*4-value
	No	%	No	%	No	%				
New or recurrent							0.193	0.147	0.811	0.092
New	17	48.6%	23	65.7%	16	45.7%				
Recurrent	18	51.4%	12	34.3%	19	54.3%				
Previous treatment							0.029*	0.218	0.028*	0.120
No treatment	17	48.6%	23	65.7%	16	45.7%				
Cryo-therapy	8	22.9%	5	14.3%	2	5.7%				
Electro-cautary	5	14.3%	3	8.6%	5	14.3%				
Keratolytics	2	5.7%	4	11.4%	11	31.4%				
Surgery	3	8.6%	0	0.0%	1	2.9%				
Duration of wart (months)							0.404	0.296	0.742	0.221
Mean ± SD	14.49 ± 13.80		13.00 ± 13.10		15.69 ± 13.14					
Median (Range)	12 (3–72)		10 (3–60)		12 (3–48)					
Site of all warts							0.375	0.970	0.309	0.174
Face & neck	10	28.6%	11	31.4%	10	28.6%				
Hand	12	34.3%	12	34.3%	7	20.0%				
Planter surface of foot	10	28.6%	10	28.6%	10	28.6%				
Dorsum of foot	3	8.6%	2	5.7%	8	22.9%				
Type of wart							1.000	1.000	1.000	1.000
Common	15	42.9%	15	42.9%	15	42.9%				
Plantar	10	28.6%	10	28.6%	10	28.6%				
Plane	10	28.6%	10	28.6%	10	28.6%				
Number of all warts										
< 10	16	45.7%	19	54.3%	14	40.0%	0.483	0.473	0.629	0.231
≥ 10	19	54.3%	16	45.7%	21	60.0%				
Mean ± SD	20.09 ± 24.12		19.97 ± 25.63		22.40 ± 23.04		0.777	0.846	0.600	0.506
Median (Range)	10 (1–90)		8 (1–90)		15 (1–80)					

*P*1 value: comparison among all groups

*P*2 value: comparison between Group I and Group II *P*3 value: comparison between Group I and Group III

*P*4 value: comparison between Group II and Group III

*P*-value < 0.05 is considered significant

Chi-square test, Mann–Whitney test

**Fig. 1:**
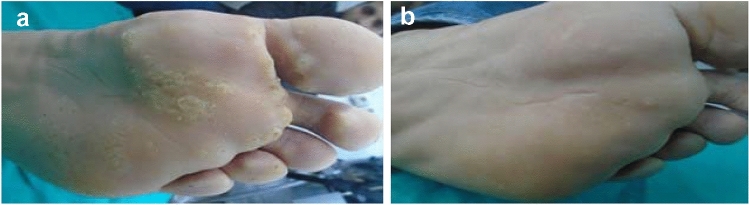
**A** Male patient, 22 years, with multiple planter warts before treatment. **B** Complete clearance after 2 sessions of intralesional Candida antigen 1/100 injection

Regarding the response to treatment, group1 (G1), treated with Candida Ag (1/100), showed that (31) patients (88.6%) had complete response, (4) patients (11.4%) with partial response and non, showed poor response (Table 2, Fig. 1).

**Table 2:** Clinical response to treatment

Response	Group I (*n* = 35)		Group II (*n* = 35)		Group III (*n* = 35)		*P*1-value	*P*2-value	*P*3-value	*P*4-value
	No	%	No	%	No	%				
Complete	31	88.6%	26	74.3%	24	68.6%	0.150	0.066	0.030*	0.868
Partial	4	11.4%	4	11.4%	5	14.3%				
Poor	0	0.0%	5	14.3%	6	17.1%				

*P*1 value: comparison among all groups

*P*2 value: comparison between Group I and Group II

*P*3 value: comparison between Group I and Group III

*P*4 value: comparison between Group II and Group III

*P*-value < 0.05 is considered significant

Chi-square test

**Fig. 2:**
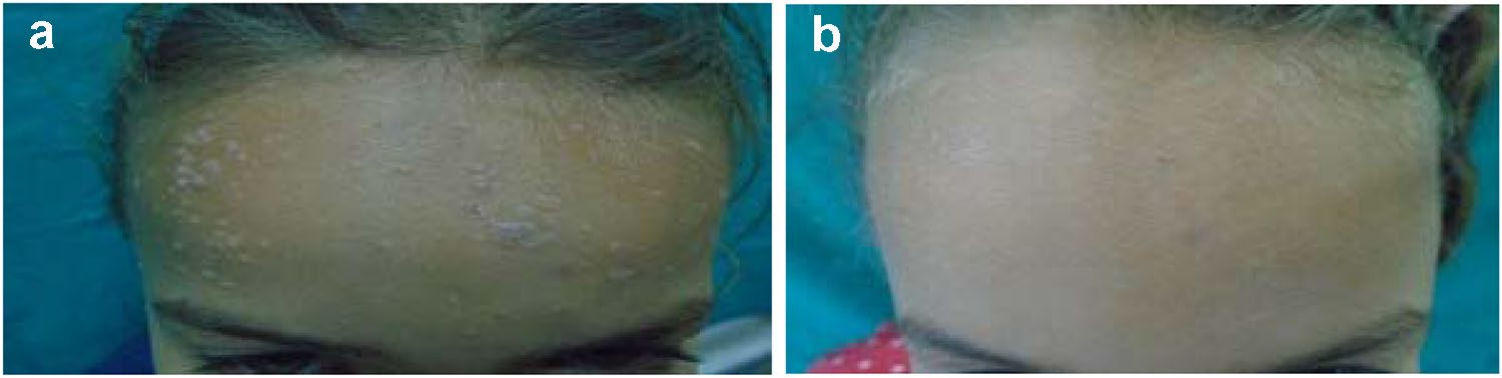
**A** Female child, 8 years, with multiple plane warts of forehead before treatment. **B** Complete response after 6 sessions of intralesional Candida antigen, 1/1000 injection.

In group 2 (G2), treated with Candida Ag 1/1000, 26 patients (74.3%) showed complete response, 4 patients (11.4%) showed partial response, and 5 patients (14.3%) showed poor response (Table 2, Fig. 2).

**Fig. 3:**
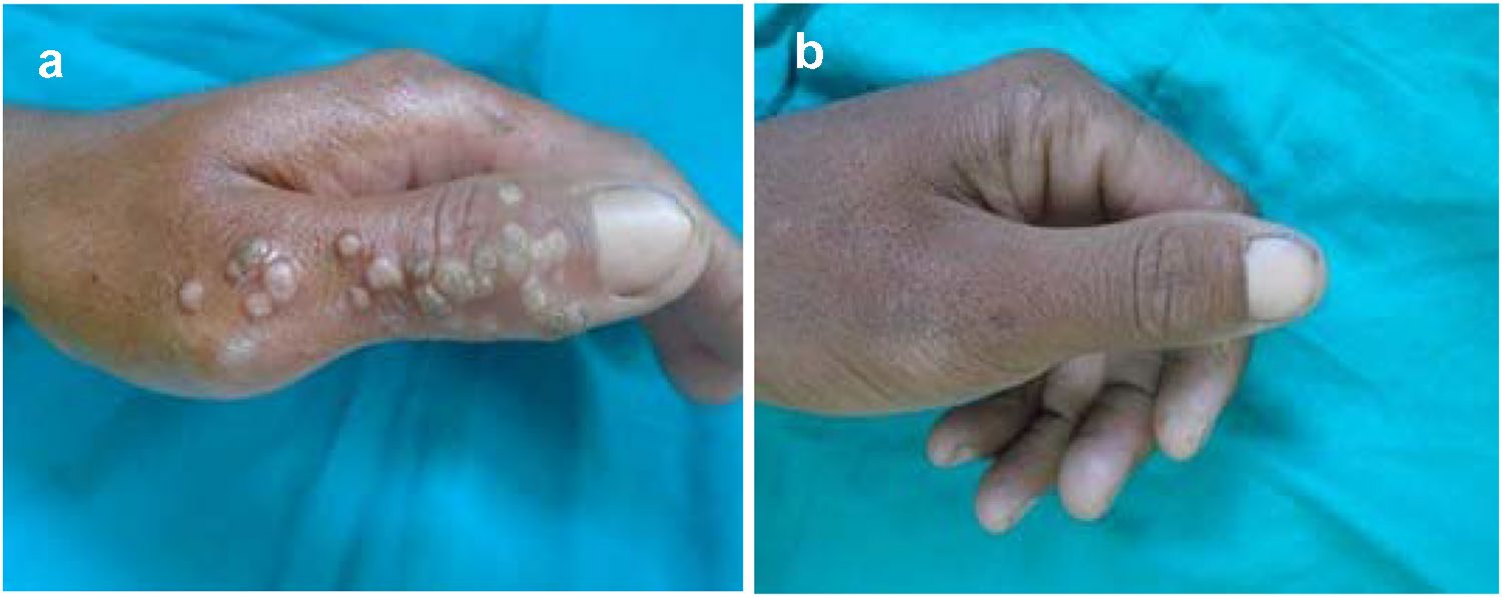
**A** Male patient, 18 years, with common warts before treatment. **B** Complete clearance after 6 sessions of intralesional Zinc sulfate 2% injection

In the third group (G3), treated with zinc sulfate, 24 patients (68.6%) showed complete response, 5 patients (14.3%) showed partial response and 6 patients (17%) showed poor response (Table 2, Fig. 3).

The highest response was detected in G1 followed by G2 and finally G3 with a statistical difference only between the first and third groups as seen in Table 2.

When comparing the treatment response of the target wart, it was found that higher rates of complete clearance were achieved in G1 (94.3%) versus G2 and G3 that showed (77.1%) and (74.3%), respectively. A statistically significant difference in response of the target wart had been detected between G1 and G3. Response of distant warts was higher in G1 in comparison to other groups with no statistical difference between them as shown in Table 3.

**Table 3:** Degree and Percentage of Improvement of Target and Distant Warts

Degree of improvement	Group I (*n* = 35)		Group II (*n* = 35)		Group III (*n* = 35)		*P*1-value	*P*2-value	*P*3-value	*P*4-value
	No	%	No	%	No	%				
Target injected wart							0.078	0.087	0.030*	0.468
Complete	33	94.3%	27	77.1%	26	74.3%				
Partial	2	5.7%	5	14.3%	3	8.6%				
Poor	0	0.0%	3	8.6%	6	17.1%				
Distant non- injected warts							0.343	0.527	0.116	0.574
Complete	29	90.6%	25	80.6%	21	70.0%				
Partial	1	3.1%	2	6.5%	4	13.3%				
Poor	2	6.3%	4	12.9%	5	16.7%				
% Of improvement										
Target injected wart							0.047*	0.033 *	0.016*	0.634
Mean ± SD	98.43 ± 6.73		87.80 ± 25.48		82.43 ± 33.20					
Median (Range)	100 (65–100)		100 (0–100)		100 (0–100)					
Distant non-injected warts							0.154	0.263	0.051	0.417
Mean ± SD	93.13 ± 24.68		85.81 ± 34.04		82.17 ± 34.13					
Median (Range)	100 (0–100)		100 (0–100)		100 (0–100)					

*P*1 value: comparison among all groups

*P*2 value: comparison between Group I and Group II

*P*3 value: comparison between Group I and Group III *P*4 value: comparison between Group II and Group III *P*-value < 0.05 is considered significant

Chi-square test, Mann–Whitney test

Regarding the percentage of improvement, there was a higher improvement in G1 in target injected wart that was statistically significant when compared to the other two groups, as shown in Table 3.

We reported that most patients were very satisfied after treatment in the three groups with no statistical difference between them as shown in Table 4.

**Table 4:** Patient satisfaction in the studied groups:

Satisfaction	Group I (*n* = 35)		Group II (*n* = 35)		Group III (*n* = 35)		*P*1-value	*P*2-valu e	*P*3-valu e	*P*4-valu e
	No	%	No	%	No	%				
Very satisfied	3 2	91.4%	26	74.3%	2 5	71.4%	0.338	0.15 2	0.13 1	0.88 6
Satisfied	2	5.7%	2	5.7%	3	8.6%				
Slightly satisfied	0	0.0%	2	5.7%	1	2.9%				
Unsatisfied	1	2.9%	5	14.3%	6	17.1%				

*P*1 value: comparison among all groups

*P*2 value: comparison between Group I and Group II *P*3 value: comparison between Group I and Group III *P*4 value: comparison between Group II and Group III

Chi-square test

Common warts showed the highest (complete) response followed by plane warts and lastly plantar warts with no statistical difference between them. Complete response was the highest and was found in most of patients either children or adults. A significant difference was found between the different degrees of satisfaction and type of response; those with complete clearance showed a great degree of satisfaction. (Table 5).

**Table 5:** Relations

	Response						*P*-value
	Complete		Partial		Poor		
	No	%	No	%	No	%	
Type of wart							0.066
Common	38	84 4	4	8.9	3	6.7	
Planter	19	63 3	4	13.3	7	23 3	
Plane	24	80 0	5	16.7	1	3.3	
Age: (years)							0.773
< 18	37	45 7	5	38.5	4	36 4	
≥ 18	44	54 3	8	61.5	7	63 6	
Satisfaction							0.000*
Very satisfied	81	97 6	2	2.4	0	0.0	
Satisfied	0	0.0	7	100 0	0	0.0	
Slightly satisfied	0	0.0	3	100 0	0	0.0	
Unsatisfied	0	0.0	1	8.3	11	91 7	
Very satisfied	81	97 6	2	2.4	0	0.0	

*P*1 value: comparison among all groups

*P*2 value: comparison between Group I and Group II *P*3 value: comparison between Group I and Group III *P*4 value: comparison between Group II and Group III *P*-value < 0.05 is considered significant

Chi-square test

Regarding the observed complication, pain was significantly higher among patients treated with zinc sulfate in comparison to the other two groups. Dyspigmentation was observed only in G3 treated with zinc sulfate in four cases. Flu-like symptoms (fever-rigors-malaise) was a significant systemic side effect reported only in both groups treated with Candida antigen (G1 and G2). Other local complications were sporadically detected in some patients in the three treated groups with no statistical difference. Recurrence had been reported in one case of G1 treated with Candida Ag 1/100 as shown in Table 6. Negative correlation had been found between both duration and diameter of warts and percent of improvement as shown in Table 7.

**Table 6:** Complications in the Studied Population

Complications	Group I (*n* = 35)		Group II (*n* = 35)		Group III (*n* = 35)		*P*1-value	*P*2-value	*P*3-value	*P*4-value
	No	%	No	%	No	%				
Pain	13	37.1%	10	28.6%	32	91.4%	0.000*	0.445	0.000*	0.000*
Swelling	6	17.1%	5	14.3%	2	5.7%	0.319	0.743	0.259	0.428
Erythema	4	11.4%	5	14.3%	0	0.0%	0.078	1.000	0.114	0.054
Peeling	2	5.7%	1	2.9%	0	0.0%	0.357	1.000	0.498	1.000
Itching	2	5.7%	0	0.0%	1	2.9%	0.357	0.493	1.000	1.000
Regional lymphadenitis	3	8.6%	2	5.7%	0	0.0%	0.230	1.000	0.239	0.493
Hematoma	1	2.9%	1	2.9%	0	0.0%	0.601	1.000	1.000	1.000
Myalgia	2	5.7%	0	0.0%	0	0.0%	0.130	0.493	0.493	–
Hypopigmentation	0	0.0%	0	0.0%	2	5.7%	0.130	–	0.493	0.493
Hyperpigmentation	0	0.0%	0	0.0%	2	5.7%	0.130	–	0.493	0.493
Flu like symptoms	10	28.6%	14	40.0	0	0.0%	0.000*	0.314	0.001*	0.000*
Headache	1	2.9%	1	2.9%	0	0.0%	0.601	1.000	1.000	1.000
No side effects	9	25.7%	6	17.1%	1	2.9%	0.027*	0.382	0.006*	0.106
Recurrence	1	2.9%	0	0.0%	0	0.0%	0.364	1.000	1.000	–

Chi-square test, Fisher Exact test

*P*1 value: comparison among all groups

*P*2 value: comparison between Group I and Group II *P*3 value: comparison between Group I and Group III *P*4 value: comparison between Group II and Group III *P*-value < 0.05 is considered significant

**Table 7:** Correlations

	Percent of improvement	
	*r*-value	*P*-value
Age (years)	− 0.164	0.094
Duration of wart (months)	− **0.327**	0.001*
Diameter of injected wart (mm)	− **0.288**	0.012*

Spearman correlation

*P*-value < 0.05 is considered significant

A statistically significant negative correlation had been found between both duration and diameter of warts and percent of improvement (in bold)

## Discussion

Intralesional immunotherapy has been successfully used in treatment of warts with good efficacy, high safety, low recurrence, and clearance of untreated warts [12]. In our study, different types of extragenital warts (common, plantar, and plane) were evident in all treatment groups. The age of patients ranged from (4 to 49 years) old. The duration of appearance of warts ranged from (3 months to 6 years) which is consistent to various studies [4, 9, 13].

In group 1, our results showed higher percentage of complete response (88.6%) compared to (60%) in *Hodeib *[10] However, this could be attributed to the more sessions done in our study (six sessions) while *Hodeib* performed only (four sessions). Also, it could be attributed to different wart type as *Hodeib* 's study was done only on verruca plana.

Our study reported complete clearance of 74.3% of patients in group 2 treated with IL candida Ag 1/1000. Similarly, *Khozeimeh *[14], reported complete remission in 76.7% of the patients. Also, *Sabry *[15] demonstrated 75.9% complete response in patients with different types of warts received IL Candida Ag 1/1000. *Muñoz Garza *[16] achieved complete response in 70.9% of patients. Nofal [17] achieved complete response in 65.7% of patients with recalcitrant planter warts. Also, *Fawzy *[13] reported complete clearance in 28 patients (70%), partial response in eight patients (20%), and no response in four patients (10%) in their verruca plana when treated by Candida Ag 1/1000.

Many previous studies supported our findings with 83.3% complete clearance as *Clifton *[18], 87% as *Maronn *[19], and even up to 100% complete remission rates as *Kim *[20].

Lower response was reported in other studies as that done by *Nofal *[9] where complete clearance was achieved in only 20 patients (55.6%), partial response in 4 patients (11.1%), and no response in 12 patients (33.3), also *Horn *[21] reported only 54% complete response in and *Alikhan *[22] with 39%, complete response.

The disagreement with the results of *Nofal *[9] may be attributed to the type of wart studied as their study was on plane warts. *Horn *[21] included recalcitrant patients, while *Alikhan *[22] reported that several patients with partial clearance were contacted via telephone, and they canceled their follow-up visit and the authors couldn't add these results only by phone. Also, several other patients stopped treatment earlier to the recommended study period (6 treatments).

In our study, complete clearance of target wart was achieved in 94.3% of G1 patients versus 77.1% in G2 and 74.3% in G3, with statistically significant difference between G1 and G3. Clearance of untreated warts including the nearby and distant lesions was an important advantage of candida Ag (1/100) as *Hodeib *[10]. It has also been reported by other studies utilizing IL Candida Ag 1/1000 injection for the treatment of different types of warts. We reported complete clearance of 90.6% distant warts in G1 patients versus G2 and G3. *Johnson *[23] reported complete resolution of the wart treated with *Candida* injections in 74% of patients and complete resolution of the untreated warts in 78% of these responders. Intralesional *Candida* immunotherapy has also been tried in children with recalcitrant warts with a response rate of 47% for the treated wart and 34% for all the body warts [18].

The exact mechanism of action of intralesional immunotherapy, including Candida Ag is still unclear [18]*.* This may be mediated through induction of strong nonspecific inflammatory response against the HPV-infected cells [24] and through an interaction of stimulated macrophages, T-helper cells, neutrophils, and natural killer cells [25]*.* Also, release of different cytokines resulting in clearance of the treated wart, and even warts at distant sites [21]*.*

Some adverse effects were observed in those who received IL Candida antigen included local reactions such as tolerable pain of the injection in all patients, edema/induration, erythema, and systemic reactions such as flu-like symptoms in a variable number of patients. However, these adverse effects were trivial, transit, and did not necessitate stoppage of treatment in any of the studied patients as was previously reported by many authors [9, 15, 18, 26].

One case of recurrence was observed after 2 months of follow-up in G1and no recurrence occurred in G2 or G3. Similar observations of absent or low recurrence rates have been reported by related studies with Candida Ag [9, 18, 21, 26–28]*.*

Zinc was proven to be used as topical or systemic oral therapy in previous studies for treatment of warts [29, 30]. Only few studies used it intralesional and showed favorable results as those done by *(Mohamed *[7]*, Moubasher *[8].

Like our results, *Moubasher *[8] reported 72.7% complete clearance in target warts and 40.9% in distant warts with IL zinc sulfate 2% injection. However, *Sharquie and Al-Nuaimy *[31] showed higher response rates, as they reported total cure rate in 98.2% of target common warts, and most of them (80.92%) needed just a single injection. This difference might be because we injected only a single wart, however, they injected multiple warts in the same session.

*Mohamed *[7] reported 88% cure rate that also was higher than ours' that may be due to type of wart treated as their study was done on only common warts that were detected to give better response with immunotherapy as we noticed in our study.

The mechanism of action of zinc sulfate is probably like the action of zinc sulfate in cutaneous leishmaniasis and bleomycin on viral warts, as both induce necrosis and inflammation. Its mechanism, as a treatment of recalcitrant warts, is not clear. It may enhance the patient's immunity via its immunomodulatory action [7]*.*

Similarly, *Mohamed *[7] reported that pain during injection was the most common complication. Also, *Sharquie and Al-Nuaimy *[31] found the side effects of this treatment were just local pain, swelling, and hyperpigmentation. We found that complete clearance was observed in most of the patients, either children or adults, regardless of their ages. Several studies stated that age had no effect on the therapeutic response in immunotherapy-treated warts, with no significant correlation was observed between age and therapeutic response [14, 32].

However, *Horn *[21] observed a significant improvement in the response for IL immunotherapy in age less than 30 years. Also, *Sabry *[15] found significant relation between treatment response and younger ages.

A significant statistical difference was found between the different degrees of satisfaction and type of response; those with complete clearance showed a great degree of satisfaction. This was against that observed by *Abd El‐Magiud *[11] who reported that there was no statistically significant difference in patients' satisfaction between the two groups treated by IL measles mumps rubella (MMR) and cryotherapy in their study.

Negative correlation had been found between disease duration and percent of improvement. In contrary to *Mohamed *[7] and *Sabry *[15] who reported that there is no significant relation found between response and disease duration. Also, we reported negative correlation between wart size and response, in contrary to *Nofal *[9] who reported that no significant relationship was found between the therapeutic response to immunotherapy and the different clinical wart variables, including, size and duration of warts.

## Conclusion

Intralesional immunotherapy with Candida antigen was more effective than iIntralesional 2% zinc sulfate in treatment of cutaneous warts and less painful. IL Candida 1/100 and 1/1000 concentrations, both were effective, well tolerable, and have nearly the same efficacy in treating cutaneous warts. Intralesional Candida Ag and IL zinc sulfate immunotherapy have significant advantages over other wart treatment modalities like single injection site and clearance of distant warts. It's simple, cheap, effective, and safe procedure with no risk of any scarring at the sites of intervention and can be used as an alternative modality in treatment of common, plane, and plantar warts either new or resistant warts.

## Data Availability

The datasets generated and analyzed during the current study are available from the corresponding author on reasonable request.
